# Isolated Ocular Manifestation of Relapsed Chronic Myelogenous Leukemia Presenting as Myeloid Blast Crisis in a Patient on Imatinib Therapy: A Case Report and Review of the Literature

**DOI:** 10.1155/2015/380451

**Published:** 2015-12-24

**Authors:** Rohit Gulati, Yaser Alkhatib, Vijayalakshmi Donthireddy, Michelle Madden Felicella, Madhu P. Menon, Kedar V. Inamdar

**Affiliations:** Henry Ford Hospital, 2799 W. Grand Boulevard, Detroit, MI 48202, USA

## Abstract

Blast phase in chronic myelogenous leukemia (CML) has rarely been reported to involve extramedullary sites like skin, lymph nodes, and central nervous system. Clinical history, characteristic hematologic findings (elevated leukocyte counts, myelocytic predominance, and basophilia), and Philadelphia chromosome are of high diagnostic significance especially in isolated extramedullary presentations. We describe a unique case of CML relapse with blast phase involving the eye. A 66-year-old man with a known diagnosis of CML on imatinib and in molecular remission for 3 years presented with a painful blind eye. Histologic examination revealed diffuse involvement of choroid, iris, vitreous humor, and the optic nerve by blast cells. The blasts expressed CD34, aberrant TdT, and a myeloid phenotype (CD13, CD33, and CD117). Fluorescence in situ hybridization (FISH) of vitreous fluid detected* BCR-ABL1* gene rearrangement. Additionally, trisomy 8 and gains of 9 and 22 were seen which were not present in the initial diagnostic marrow study 3 years ago. At relapse, the bone marrow, peripheral blood, and the cerebrospinal fluid were not involved by CML. Patient received induction chemotherapy and single dose prophylactic intrathecal methotrexate and was maintained on antityrosine kinase therapy and eventually underwent allogenic stem cell transplantation.

## 1. Introduction

Blast crisis in CML is defined by the presence of ≥20% blasts in the bone marrow or peripheral blood, large clusters of blasts in the bone marrow biopsy, or any extramedullary blast proliferation [1]. Extramedullary disease occurs in a small percent of CML cases and is found to manifest in its chronic, accelerated, or blast phase [2, 3]. It can occur at various sites including bone, lymph node, skin, soft tissue, and central nervous system (CNS) [3–6]. We describe an unusual case of relapsed chronic myelogenous leukemia (CML) in blast crisis presenting with isolated ocular involvement in a patient undergoing imatinib therapy and hematologically in morphologic, cytogenetic, and molecular remission at the time of relapse.

## 2. Case Presentation

Our patient is a 66-year-old male who was first diagnosed with CML after presenting to the emergency room with acute onset knee pain. During the investigation, he was found to have an elevated white blood cell count (64.4 × 10^9^/L). The differential count revealed neutrophilia with left shift and basophilia. A subsequent bone marrow biopsy showed morphologic features consistent with chronic phase of CML. Conventional karyotyping and fluorescence in situ hybridization (FISH) on the bone marrow aspirate (Figure 9) showed evidence of t(9;22)* BCR-ABL1* rearrangement and real time quantitative PCR was positive for* M-BCR/ABL* t(9;22), p210 fusion transcript. The patient was started on imatinib 400 mg once a day. He achieved major molecular response after 3 months of therapy and was followed up every 3-4 months thereafter with* M-BCR/ABL* [t(9;22), p210] fusion gene transcript levels in peripheral blood by real time quantitative PCR.

He remained in remission for three years after initial diagnosis, when he presented with sudden onset pain and loss of vision in the right eye. Magnetic resonance imaging (MRI) scan showed significant intraorbital and optic nerve enhancement. An infratemporal intraconal fat and intraorbital fat biopsy was negative for malignancy. The patient's symptoms improved with conservative management by posterior chamber decompression and steroid therapy but his vision never returned to baseline following this episode. A follow-up MRI 2 months later showed no residual signs of enhancement. Eight months following this episode, he presented with recurrent pain in the right eye, which did not resolve with conservative management. As a result, the patient underwent enucleation of his right eye.

**Figure 1:**
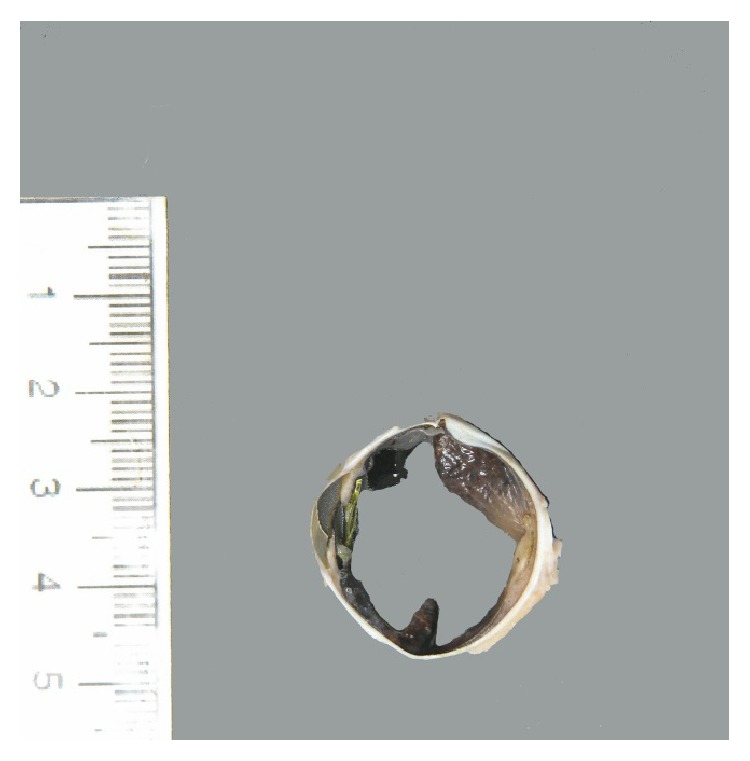
Gross photomicrograph depicting pupil-optic nerve (PO) section of enucleated eye which reveals diffuse thickening of the iris and part of the choroid layer.

**Figure 2:**
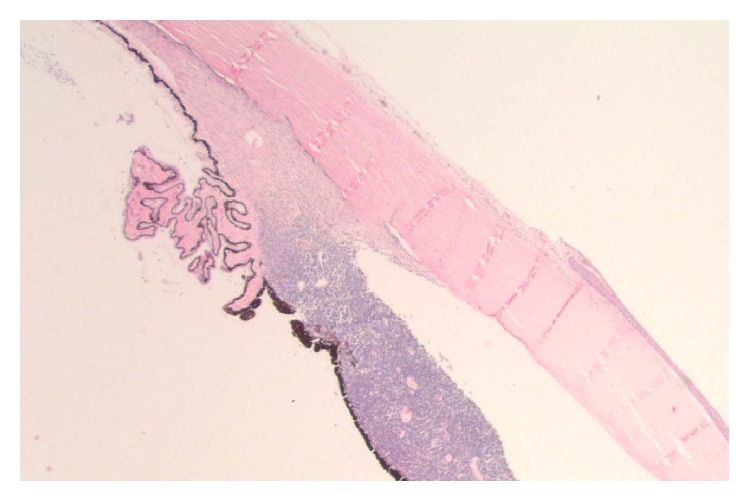
Blasts cells infiltrating the iris (H&E, magnification ×20).

**Figure 3:**
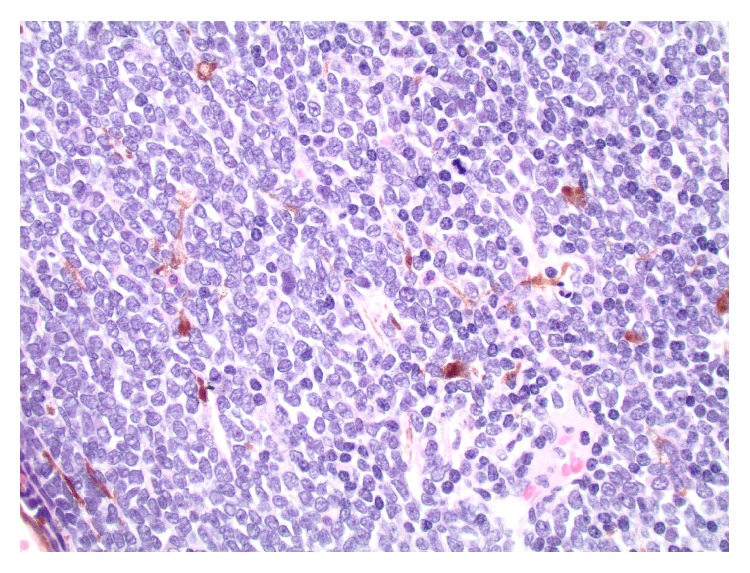
Sheets of monotonous mononuclear blast cells (H&E, magnification ×400).

**Figure 4:**
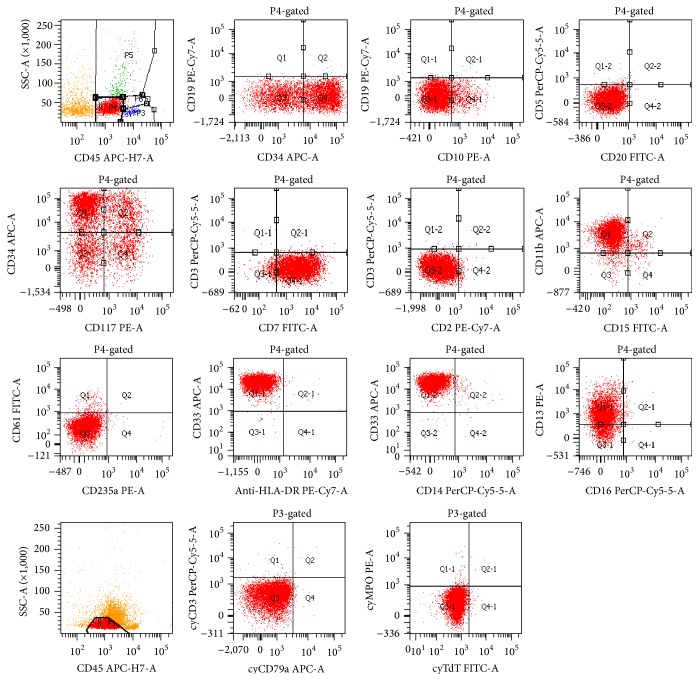
Flow cytometric immunophenotypic analysis of vitreous fluid: blast gate (P4 gate in the top line) represents the dim CD45+ cells with low side light scatter (SSC). Blast gate (P3 gate in the bottom line) represents the same population of blasts analyzed for cytoplasmic markers in a separate tube. The distinct cluster of blasts (red color population) exhibit the following immunophenotypic characteristics:* positive for* CD34, CD117, CD7, CD11b, CD13 (dim), and CD33;* negative for* CD19, CD10, CD20, CD3, CD5, CD15, CD16, CD61, CD235a, HLA-DR, CD14, cytoplasmic CD3, cytoplasmic CD79a, cytoplasmic MPO, and cytoplasmic TdT.

**Figure 5:**
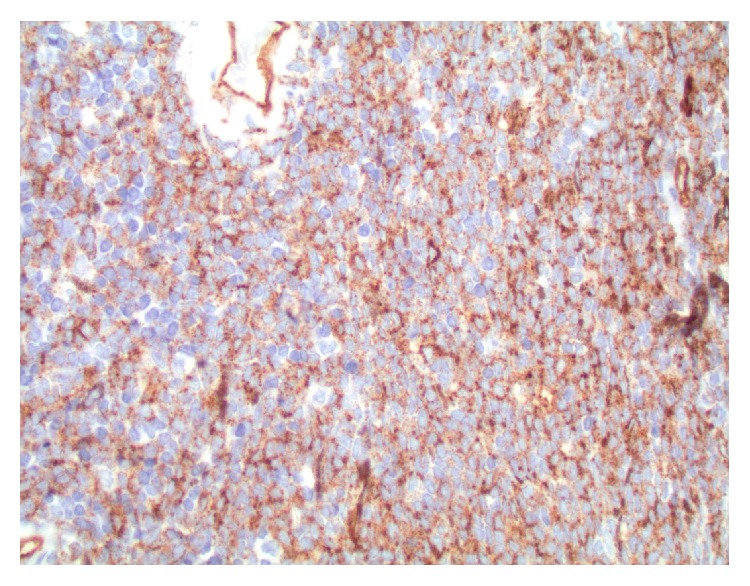
CD34 immunohistochemistry: sheets of monotonous CD34 positive blast cells (magnification ×400).

**Figure 6:**
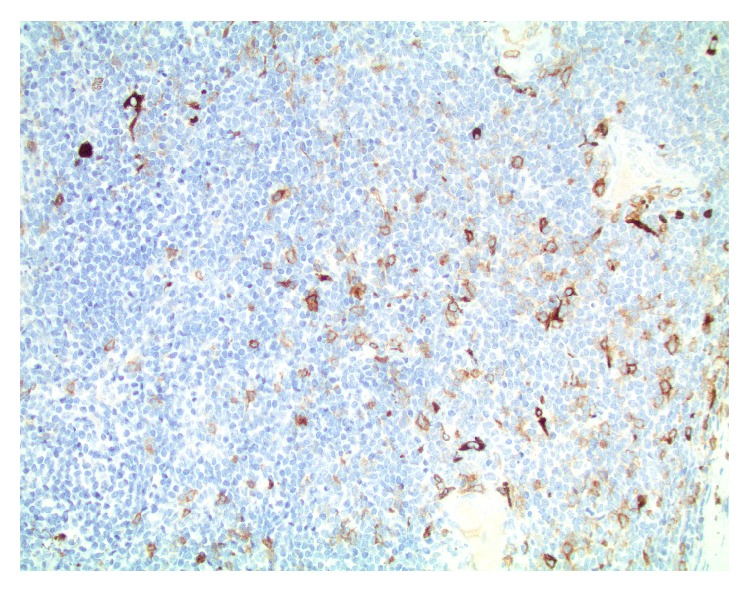
CD117 immunohistochemistry: focal and variable intensity of CD117 expression is seen in the blasts (magnification ×400).

**Figure 7:**
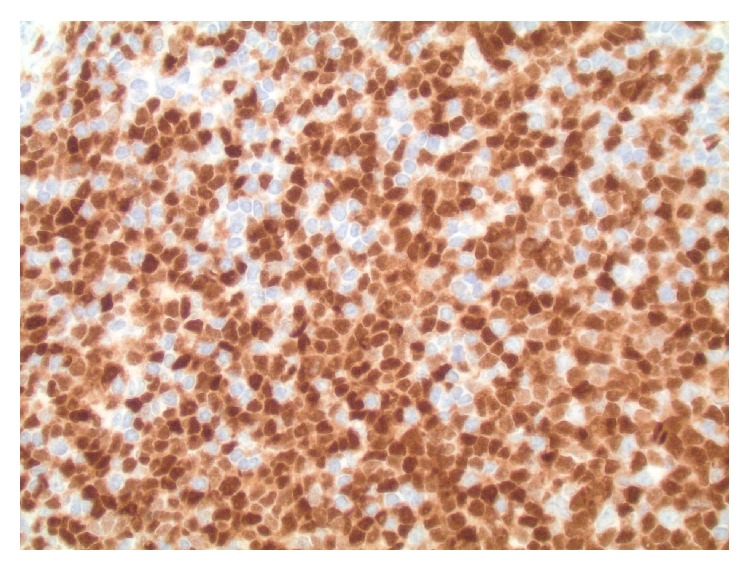
TdT immunohistochemistry: sheets of blasts showing nuclear expression of TdT (magnification ×400).

**Figure 8:**
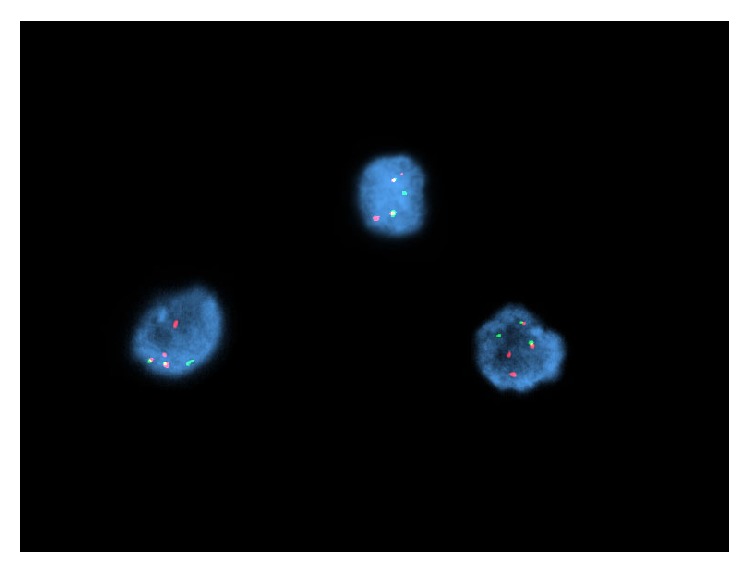
Fluorescence in situ hybridization assay on vitreous humor smear shows two BCR-ABL1 fusion signals, one BCR probe signal, and two ABL1 probe signals; thus a t(9;22) BCR-ABL1 gene rearrangement with gains of 9 and 22 [orange = 9q34 ABL1 probe; green = 22q11.2 BCR probe].

**Figure 9:**
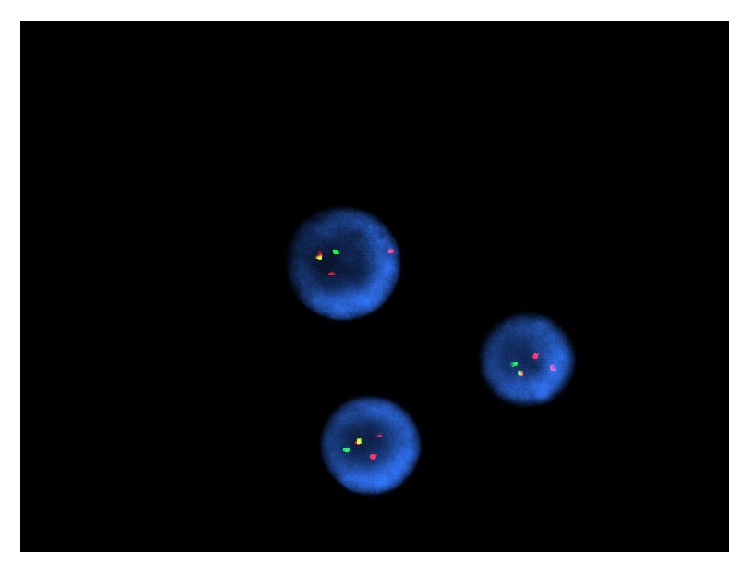
Fluorescent in situ hybridization on original diagnostic bone marrow aspirate shows one BCR-ABL1 fusion signal, two BCR probe signals, and three ABL1 probe signals in 90.5% of interphase cells.

On gross examination, a cross section of the eye showed diffuse circumferential thickening of the choroid (Figure 1). Microscopic examination revealed dense infiltration of the choroid and iris and optic nerve margin by a monotonous mononuclear cell infiltrate morphologically resembling blasts (Figures 2 and 3). By flow cytometry (Figure 4), the blasts were positive for CD34 and demonstrated myeloid immunophenotype expressing CD13, CD33, and CD11c. Blasts were negative for specific lymphoid markers including CD19, CD20, CD79a, CD2, cytoplasmic CD3, CD5, and CD10. However, CD7 was aberrantly coexpressed by the blasts. Interestingly, the blasts did not show cytoplasmic myeloperoxidase (MPO) and terminal deoxynucleotidyl transferase (TdT) expression by flow cytometry. By immunohistochemical staining (Figures 5–7), the blasts were positive for CD34 (Figure 5) with focal expression of variable intensity for CD117 (Figure 6) and strong and diffuse expression of TdT (Figure 7) but were negative for B- or T-lineage lymphoid antigen markers (CD20, CD3, and CD79a) (not shown). MPO expression was focal and weak (not shown). FISH cytogenetic analysis on vitreous fluid (Figure 8) demonstrated presence of* BCR/ABL1* [t(9;22)] gene rearrangement identical to the abnormalities detected in the original bone marrow (Figure 9) in which the diagnosis of CML was rendered. In addition to Philadelphia chromosome (Ph), a complex karyotype including trisomy 8 and gain of chromosomes 9 and 22 was also detected in this analysis. In view of the patient's history, a diagnosis of CML in blast phase was rendered. A subsequent bone marrow exam showed no morphologic, cytogenetic, or molecular evidence of CML or acute leukemia. Cytological evaluation and flow cytometry of cerebrospinal fluid were also negative for disease involvement. The patient underwent induction therapy with idarubicin and cytarabine and a single dose of intrathecal methotrexate. He was maintained on dasatinib 150 mg daily but was switched back to imatinib due to intolerance to dasatinib. He subsequently underwent allogenic stem cell transplantation after 5 months.

## 3. Discussion

Extramedullary blast crisis in CML is a rare occurrence. In a study of 235 CML patients by Specchia et al. [2], 16% developed extramedullary blast crisis. While both myeloid and lymphoid blast transformations are described, 80% of transformations are of myeloid origin, whereas lymphoblastic transformations account for approximately 20%. Among the latter, B-lymphoblastic transformations are more common than T-lymphoblastic crisis [4, 7]. A recent study looking at outcomes found no significant difference in overall survival of CML in myeloid versus lymphoid blast crisis [7]. Ocular manifestations as presenting feature of CML have been previously described. The first case report of ocular involvement by CML in blast phase was published by Alegre et al. [8] but the patient had evidence of disease at other sites. Isolated ocular blast crisis was first described by Lipton et al. [9], who reported a case of relapsed CML presenting as lymphoid blast crisis. In their patient, lymphoid blast crisis was also the initial presentation of the disease at the time of diagnosis. Conversely, our patient initially presented in chronic phase of CML and relapsed with myeloid blast crisis in the eye. Immunophenotypically by flow cytometry, the blasts expressed CD34, CD13, and CD33 with a subset expressing CD117 whereas they were negative for cytoplasmic MPO, TdT, and B- (CD19, CD22, CD79a, and CD10) as well as T-lymphoid antigen markers (cytoplasmic CD3). In contrast to flow cytometry, the blasts were strongly and uniformly positive for TdT by immunohistochemistry. The reason for this discrepancy is not entirely clear but we speculate that the lack of TdT expression by flow cytometry may be attributed to failure of permeabilization step in the protocol for assessment of cytoplasmic markers. The lack of expression of lymphoid markers and positivity for CD13, CD33, and CD117 was consistent with myeloid immunophenotype for the blasts.

In our case, the patient was treated with cytarabine/doxorubicin and intrathecal methotrexate. Imatinib mesylate is considered first-line therapy for treatment of CML in chronic phase with majority of patients achieving complete cytogenetic and major molecular response. Sudden evolution of disease into blast phase can rarely occur while the patient is on imatinib therapy [10]. Isolated CNS involvement by CML in blast phase has been reported in patients receiving imatinib mesylate treatment [11, 12]. It usually presents as a relapse following an initial complete remission on imatinib or after bone marrow transplant. In some patients, there may be no signs of disease involvement in the blood and bone marrow at time of CNS manifestation [12]. The central nervous system is relatively prone to disease involvement while on imatinib therapy and this is attributed mainly to poor permeability of imatinib through the blood brain barrier [11, 13]. The most common sites of CNS involvement are the lateral ventricular walls [14], meninges [15], and the optic nerve [16], causing symptoms such as headache, cognitive changes, raised intracranial pressure, and visual disturbances. Intraocular optic nerve involvement has been associated with poor prognosis and impermeability to intrathecal chemotherapeutic agents [17]. Increasing reports of CNS involvement by CML in different phases have necessitated broader disease monitoring, including BCR-ABL gene quantification in CSF [14]. Second-generation tyrosine kinase inhibitors (TKI) such as dasatinib are more effective in treating CNS disease [18] due to better penetration of the blood brain barrier [19]. For these reasons, dasatinib and nilotinib are now FDA-approved as first-line therapy in CML [20].

In our patient, the chromosome study was positive for t(9;22)(q34;q11) translocation, FISH showed* BCR/ABL* fusion gene, and RT-PCR assay was positive for major* BCR-ABL* mRNA expression. His previously documented diagnosis of chronic phase CML was helpful in diagnosing his ocular blast proliferation/myeloid sarcoma as a blast phase of CML rather than a genetically different de novo acute myeloid leukemia [4, 21, 22]. While blast proliferation in an extramedullary setting would be most consistent with a diagnosis of myeloid sarcoma, since our patient had a documented history of CML, we preferred to use the term blast phase of CML to guide therapeutic decisions. While t(9;22)(q34;q11) translocation is most commonly associated with CML or precursor B-ALL, its presence can be rarely seen in acute myeloid leukemia with a reported incidence of 1-2% of all AML cases. In the absence of a prior chronic or accelerated phase of CML, distinction between blast phase of CML and Philadelphia chromosome-positive (Ph+) AML can be difficult, as they exhibit a major overlap in their clinical presentation and genetic features [23]. Nevertheless, presence of splenomegaly, basophilia, and chromosomal abnormalities in addition to Philadelphia chromosome and a previous history of chronic or accelerated phase CML have all been attributed to blast phase of CML over Ph+ AML [24, 25].

At the time of relapse, in addition to t(9;22)(q34;q11) reciprocal translocation (Ph+), our patient demonstrated other chromosomal aberrancies including trisomy 8 and gains of chromosomes 9 and 22. Trisomy 8 has been frequently associated with clonal evolution in CML patients on imatinib who are otherwise in complete cytogenetic remission [26]. At the genetic level, trisomy 8 has been linked to alterations of* MYC* located at 8q24 [26]. Interestingly, no* MYC* alterations were detected in our patient. Gains of chromosomes 9 and 22 appear to be nonrandom chromosomal aberrations and perhaps contribute to overall genomic instability; however, the mechanisms of clonal evolution in CML are not well understood.

Using whole genome sequencing, Calabretta and Perrotti [26] found mutations in 9 genes, including GATA2, MAX, ENO1, and ANO5, which were linked to progression of CML to blast phase. Gain-of-function mutations in GATA2 were described in a previous study which reported myeloid blast transformation in CML [27].* MAX* gene, through its complex interactions with* MYC* [28] and* CEBPA* [29], has been linked to tumor progression [30].* ENO1* acts as a transcriptional repressor of* MYC* [31]. Recently it has been shown that transcription regulators, including Hes1 and Evi1, are involved in disease progression and even confer resistance to tyrosine kinase inhibitors [32, 33]. Many recent studies have identified potential markers as a means of better monitoring of disease progression.* SYK* gene activation is one such potential marker of disease progression and dasatinib resistance [34, 35].

## 4. Conclusions

While extramedullary manifestation of CML is rare, a high degree of suspicion is warranted in patients with CML on imatinib therapy who manifest CNS or ocular symptoms, even in the absence of any hematologic, cytogenetic, or molecular evidence of disease. Despite advances in molecular disease detection, more sensitive and effective methods of monitoring disease progression are still needed.

## Abbreviations

CML: Chronic myelogenous leukemia
FISH: Fluorescent in situ hybridization
CNS: Central nervous system
PCR: Polymerase chain reaction
MRI: Magnetic resonance imaging
FDA: Food and Drug Administration.
